# Cognitive sequelae of COVID-19, a post-pandemic threat. Should we be worried about the brain fog?

**DOI:** 10.1590/0004-282X-ANP-2022-E003

**Published:** 2022-03-30

**Authors:** Oscar H. Del Brutto

**Affiliations:** 1 Universidad Espíritu Santo, School of Medicine, Research Center, Samborondón, Ecuador. Universidad Espíritu Santo School of Medicine Research Center Samborondón Ecuador

The Coronavirus Disease 2019 (COVID-19) pandemic caused by the Severe Acute Respiratory Syndrome Coronavirus-2 (SARS-CoV-2) has affected 438 million people as of February 2022, and is expected to affect more individuals during the upcoming years. It is likely that millions of COVID-19 survivors will be left with chronic sequelae of this disease. Indeed, the term “long COVID” has been coined to define the persistence of clinical manifestations or laboratory abnormalities that persist beyond the acute phase of COVID-19^1^. One of these manifestations, namely, cognitive decline, has been colloquially defined as “brain fog” due to difficulties in concentration and deteriorations in memory, receptive language and executive functions reported by a sizable proportion of COVID-19 survivors^2^. 

A recent systematic review identified more than 50 long-term effects of COVID-19, many of them related to the compromise of the central nervous system^3^. The most important neurological sequelae include cognitive impairment, sleep disturbances, headache, sarcopenia, fatigue, hearing loss, ageusia and anosmia^3^^,^^4^. Some of these sequelae not only occur in patients with a severe initial disease but in those who only experienced mild COVID-19, and may persist for several months after the acute episode^5^^,^^6^. 

Preliminary reports anticipated the devastating consequences that SARS-CoV-2 would have in developing countries^7^. Poverty, along with illiteracy and health inequalities, were pointed out -among other factors -as responsibles for this gloomy scenario. More than two years after the start of the pandemic, information is still limited on the situation of these populations. However, available data suggest that once the virus is introduced into a given population, it spreads rapidly and infects a significant proportion of the population^8^. 

Long COVID often manifest several weeks or months after the infection and do not necessarily occur in patients with complicated disease requiring hospitalization during the acute phase of the infection. Little is known about mechanisms underlying late neurological sequelae of SARS-CoV-2 infection. It has been postulated that the virus itself may be the cause of this type of sequelae in patients with long COVID^9^. The portal of entry of SARS-CoV-2 to the nervous system is often the nasal epithelium that contains fibers of the olfactory nerve. The virus then travels to the olfactory bulb and spreads by trans-synaptic transfer to limbic structures and subsequently to deeper parts of the brain^10^. An alternative hypothesis suggests that abnormalities in brain metabolism related to inflammatory or autoimmune mechanisms may play an important role in the occurrence of late sequelae^11^. This is supported by the finding of inflammatory biomarkers in patients with COVID-19-related encephalopathy, which develop a few weeks after disease onset, together with the absence of detectable levels of SARS-CoV-2 by RT-PCR in the cerebrospinal fluid^12^. 

The study of Crivelli and co-workers, published in this issue of *Arquivos de Neuro-Psiquiatria*, is a welcomed addition to the limited information on cognitive sequelae of long COVID in Latin America^13^. The investigators recruited a sample of COVID-19 survivors without apparent evidence of cognitive complaints before the pandemic and compared them with a similar number of non-infected individuals matched for age, sex, and education levels. Using a complete battery of neuropsychological tests, which was performed a mean of 5 months after a clinical episode of SARS-CoV-2 infection, it was noted a significant difference across groups in regards to memory, attention, executive functions and language. Of interest, a complete neuropsychological assessment was needed to identify such abnormalities, which were not evident by the use of commonly used tests for rapid cognitive assessment (Mini Mental Status Examination or the Montreal Cognitive Assessment). 

As the authors correctly noticed, the small sample size together with the lack of formal cognitive assessment before the pandemic and the biased selection of study participants are limitations of their study. Nevertheless, these limitations are counterbalanced by the extensive neuropsychological assessment and the matched selection of control subjects. In this view, the study provides another piece of evidence suggesting the presence of cognitive decline some months after the infection. The aforementioned study -as well as other reports -do not allow the estimation of the pathogenesis of cognitive complains among COVID-19 survivors nor the length of this complication.

Some recent studies have seized on the pathogenesis of long-COVID-related cognitive complains, which may also be of value to estimate the length of these complications. In one of these studies, it was demonstrated that COVID-19-related cognitive complaints may persist after one year of follow-up, particularly in women and in subjects with increased antinuclear antibodies titers, suggesting that autoimmunity may be responsible for this sequelae^14^. Another study revealed that high levels of C-reactive protein were related to persistence of cognitive impairment after seven months of follow-up^15^. Therefore, it seems reasonable to support the occurrence of a paucisymptomatic initial inflammatory response that express months after the acute disease leading to a dysregulated immune response, which in turn, lead to cognitive decline that improves when the inflammation subsides. 

A recent study from our group -that included community-dwelling middle-aged and older adults evaluated before and after the pandemic -revealed that most COVID-19 survivors who developed cognitive decline six months after the infection, spontaneously improved one-year later^16^. This suggested that the “brain fog” may dissipate over time. While this information is still preliminary and need to be confirmed in subsequent (and larger) cohorts, study results provide hope to millions of people suffering cognitive complains after an acute COVID-19 episode. In the meantime, it seems prudent to periodically repeat cognitive assessments in individuals complaining long-COVID-related cognitive decline and to administer proper interventions for these patients.
